# Supplementary material for: Learning curve and surgical outcome of robotic assisted colorectal surgery with ERAS program
Source: Sci Rep. 2022 Nov 29;12:20566. doi: 10.1038/s41598-022-24665-w (PMC9709162; doi:10.1038/s41598-022-24665-w)
Supplement: Supplementary file 2 — Supplementary Information 2. [file 41598_2022_24665_MOESM2_ESM.zip › RAL Raw data-Ñ[▒K/069/OP_ñw╝╨░O▒Kñσ.pdf]

正式摘要(電子簽章：000000213951006)

W55 / W55

臺中榮民總醫院

Operation Note

[redacted]

- 手術日期：2021/04/01
- 手術醫師：VS 林俊余，CR 楊曙榮，CR 林陳彥，R 陳智翔
- Anesthesia : GE
- Position : Lithotomy
- Aseptic agents : Soap , Betadine Alcohol , Betadine Aqua

Diagnosis

- Pre-operative diagnosis : Malignant tumor of anal canal and anus. APR
- Post-operative diagnosis : Ditto.

Surgical Indication

- For tumor resection

Operation

- Robot Assisted APR

Finding

- 1. anal canal tumor
  2. Left inguinal LN enlarge

Procedure

- A ROBOTIC abdominal perineal combine resection were done with general anesthesia, with lithotomy position, foley catheter in place. We makes several incision in the lower abdomen to insert the trocar. The position of trocar placement was shoen below. The IMA root was identified and skeleton by hemolock initially. Then IMV was also ligated in the same level. After retroperitoneal dissection , the left side colon and rectum was modilized and free from the retroperitoneum, pelvis. Then we dissect the left side pelvic LN carfully. Then the GU dissect the inguinal LN. Then the anus was resect and dissect. The specimen from signoid colon to the anus was retracted from the perineal wound with caution. After check bleeders mediculously, the penrose drainage tube was placed in the low pelvis. The fascia layer and skin was closed by 1-0 vicryl and 3-0 Nylon. The end-colostomy was create at the LLQ. He stood the whole procedure well and sent to POR under stable condition.
- Estimated blood loss: minimum
- 器械數量: 正確
- 紗布數量: 正確

Image

- PAINT
  - abdomen

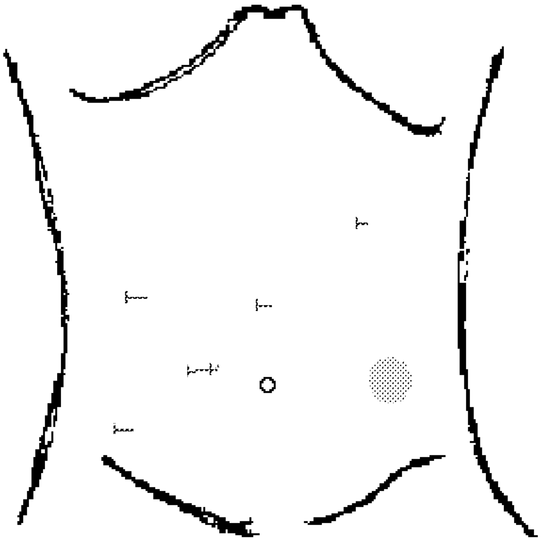

○ colon & rectum

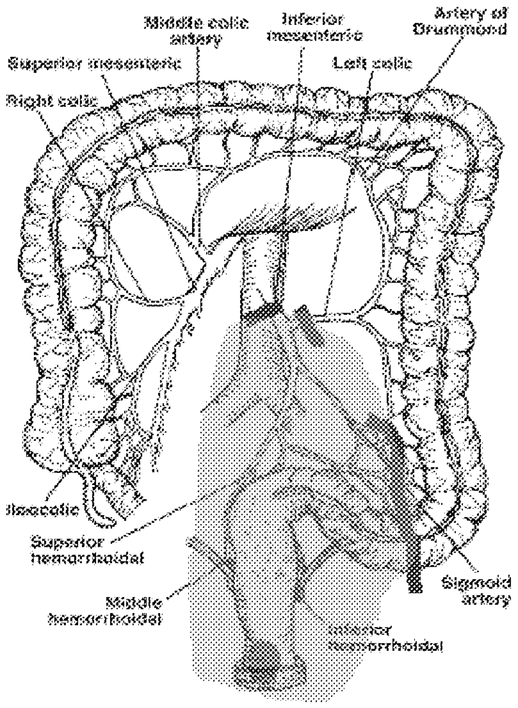

主治 2222H林俊余 / 醫師 2292G楊曙榮

正式摘要(**電子簽章：000000213951006)**

W55 / W55

doctorRank:B
